# Etiology of Diabetes

**Published:** 1858

**Authors:** G. Owen Rees


### Etiology of Diabetes.

BY DR. G. OWEN REES.

*Diabetec Sugar not the same as the Sugar produced in the Liver in Health.*—Dr. G. Owen Rees, in his valuable Croonian lectures, recently delivered before the Royal College of Physicians, makes the following interesting remarks on this subject—

According to M. Bernard, we have not now to determine how a substance, foreign to the healthy constitution of the blood, becomes engendered in the system, but merely to inquire into the causes producing, on the one hand, an over-activity in the sugar-forming action of the liver, or, on the other, the diminution of the destructive power apparently possessed by the blood in health over that sugar when it has mingled with the circulating fluid.

Now, all this is clear enough were the sugar secreted by the liver, and that produced by injuring the base of the fourth ventricle, identical with that existing in the urine of true diabetes. This, however, is not the case, and we are not, therefore, so nearly about to unravel the difficulty as we might, at first be inclined to believe.

About two years ago I took the opportunity of obtaining blood from the hepatic veins of a dog, in order to determine the presence of sugar; for, like many others, I was, at first, a little incredulous. By the assistance of my friend, Mr. Hilton, this was effected without much difficulty.

On examining the blood obtained in this way, I found, it is true, that it yielded me sugar; but there was a peculiarity in the reaction of the tests, which led me to suspect I was not dealing with the same sugar as that contained in the urine of diabetes.

It was quite impossible for me at the time to undertake a chemical investigation of the subject, and I was not sufficiently satisfied with my results to venture on publication. Some months ago I mentioned my suspicions to my friend, Dr. Pavy, who has thrown much light on this interesting subject, and he told me that the same doubt had occurred to him some time since, and he immediately showed me from his note-books that he had worked the question out very satisfactorily, though he had not published on the point. Having Dr. Pavy's permission to do so, I will now detail the results of his investigations. It appears that the principal point of difference between these sugars consists in the greater facility possessed by the hepatic sugar, and by the sugar of *artificial* diabetes, of undergoing destruction by contact with animal tissue. This has been shown by an experiment made on the sugar of *artificial* diabetes, comparing the result with that obtained by similarly treating grape sugar and *true* diabetic sugar. The experiments were conducted as follows:—Three vessels were taken. In the first a quantity of pounded liver, obtained from a healthy dog, was placed with a solution of the urine of artificial diabetes; the specific gravity of the solution was 1045. In the second vessel was placed pounded liver with a solution of common grape sugar, of specific gravity 1040. In the third was placed pounded liver with a solution of extract of true diabetic urine, of specific gravity 1040. The pounded liver was used, (as any other animal matter might have been,) merely to induce changes in the elements of these saccharine principles by its presence. The three mixtures were then set aside for nine days. At the end of that time, on submitting them to examination by Barreswil's solution, it was found that the artificial diabetic sugar had entirely disappeared, while the reactions were obtained in all their completeness from the two other solutions. Experiments made with the same solutions, substituting blood for pounded liver, led to the same results, showing a power of resisting decomposition on the part of grape sugar and *true* diabetic sugar far exceeding that existing in sugar obtained by the production of diabetes artificially.

There seems little doubt that the sugar of diabetes is a higher quality of the principle, and that it can preserve its atomic arrangement with far greater force than the hepatic variety. A power, however, seems to reside in the blood, which, after *some length of time*, eventually destroys, not only hepatic sugar and that of diabetes artificially produced, but even that of true diabetes mellitus. Thus, Dr. Pavy's experiments show that if the blood taken from a diabetic be allowed to coagulate, and the serum then be separated from the crassamentum, we can detect scarcely any evidence from the latter after a very long exposure. In the serum, however, it can be detected in quantity till decomposition is thoroughly set in. For some considerable time, both crassamentum and serum give full evidence, however, which contrasts strongly with the reaction of blood taken fresh from the right ventricle in health, and which contains *hepatic* sugar, for here the sugar disappears almost immediately the separation into serum and clot is completed. It is almost certain that when we produce the artificial diabetic state, by operation, we obtain in the urine the hepatic sugar of the liver. It is also proved that this sugar of *artificial* diabetes is not the same as the sugar of *true* diabetes.

Now, of course, were these sugars identical, we might consider true sacharine diabetes as a disease in which the sugar-forming property of the liver became abnormally active; or, on the other hand, a disease in which normal sugar was formed in the liver in usual quantity, but that the blood had lost the power of destroying it when so formed, and that it, therefore, appeared in the urine.

The results I have detailed place us, however, in a very different position. We know now that true diabetic sugar is destructible only with great difficulty, and that it is not the same as ordinary hepatic sugar. The question will then arise—Are we to regard the sugar of diabetic urine as a modification of that poured into the blood by the hepatic veins in health, or, on the other hand, as a product of disease bearing no relation whatever to the sugar of the liver?

To those who have studied the subject of sugar, in its chemical relations, who are acquainted with its varieties, and the facility with which these are convertible into each other, by the most simple processes, there will be no difficulty in believing that the sugar of diabetes may be easily derived from that produced in the liver in health. Late experimenters on the sugars obtained from the vegetable kingdom, have shown how easily transmutations are thus effected, and chemical properties developed or abstracted by simple contact with materials apparently possessing anything but chemical activity. No one can fail to be struck, for instance, with the curious fact that the sugar contained in fruits possesses a certain action on light, influencing polarization, which action is precisely reversed in the sugar obtained *by crystallization* from the very same source. Thus, the gummy kind of sugar obtained from grapes possesses the property of *left-handed* circular polarization; but if we allow this sugar to lie exposed, a kind of imperfect crystallization occurs throughout the mass; and if we collect the granular crystals so formed, we find we have in these a sugar differing materially from that originally extracted from the fruit. Its chemical constitution is not the same. Its constitution is $C12\ H14\ O14$, instead of $C12\ H12\ O12$; and when examined optically, it is found to possess the property of *right-handed* circular polarization. The change appears to be effected here by some constituent of the vegetable juice exercising its influence as crystallization goes on—probably the acids play an important part. Now, the liver, owing to some diseased action, may be supposed, in diabetes, to produce a sugar differing from that of health—a sugar which cannot be destroyed by the changes taking place naturally in the blood—changes rapidly affecting and destroying healthy hepatic sugar.

The phenomena of diabetes mellitus are, then, not quite so simple as the experiments and discoveries of Bernard would, at a first view, make them appear; and we have yet to determine the causes in action for the formation of this abnormal sugar. Does the presence of a different ferment interfere—even as we observe catalysis productive of varying results out of the body—may not an analogous action be going on in the liver? and, if so what may be the nature of the ferment productive of disease, and whence is it derived? Are we to look to the portal blood for the ferment, or controlling influence which forms this less-destructible sugar? And is it owing to this diseased state of blood that the liver, even though unaffected, is unable to cause the changes occurring in health?

But we need not have recourse to the theory of a ferment. The portal blood may present such principles to the liver as are only convertible into the *true diabetic sugar.* So far as we can yet determine, then, the whole phenomena of diabetic disease may eventually be traced to an abnormal state of the bile, gastric juice, and pancreatic secretion, any one or all of which may interfere with the formation of healthy products in the portal blood, and so overpower a healthy liver in the discharge of its office. Analogy would certainly, however, rather direct us to conclude that in diabetes the function of the liver becomes altered under the influence of some cause as yet unknown. Bernard has proved that the organ in health has a very strong transformative action on grape sugar; and so powerful is this, that we should almost be entitled to conclude, even in the event of the portal blood bringing diabetic sugar, ready prepared, into the hepatic circulation, that it would be metamorphosed by the liver into normal *hepatic* sugar before it could reach the cava through the hepatic veins.

These results, then, taken together, render it probable that we are to look for the cause of diabetes mellitus in a disturbed state of the hepatic function, not in an increase of *natural* action, but in an action varying in *kind*. We see that in health the liver would reduce proximate animal principles to a normal hepatic sugar, and, in the perversion of force occurring in diabetes mellitus, we have a product given us approaching in character, it is true, to the normal sugar, but by no means identical with it. There is great facility for theorizing with respect to the agencies in operation in affecting this change of action. As vegetable juices contain principles which, by simple contact, can alter the chemical and optical qualities of sugar first generated in the fruit, how easy to believe that the elaborate fluids contained in the several parts of the circulatory system of the liver may do the same. We know that acids are active in the vegetable kingdom—we know that the liver substance is acid—may not an over acid state cause the production of this abnormal sugar? or may not even a too slow circulation through the organ (by allowing too long contact with acid matter) bring about disease? These are questions requiring much consideration."—*Lancet*, May 30, 1857.

